# Plastic neo-vaginal construction in Mayer-Rokitansky-Küster-Hauser syndrome: an expert opinion paper on the decision-making treatment process

**DOI:** 10.3205/iprs000087

**Published:** 2016-02-03

**Authors:** Luz Angela Torres-de la Roche, Rajesh Devassy, Sreelatha Gopalakrishnan, Maya Sophie de Wilde, Anja Herrmann, Angelika Larbig, Rudy Leon De Wilde

**Affiliations:** 1 Clinic of Gynecology, Obstetrics and Gynecological Oncology, University Hospital for Gynecology, Pius-Hospital Oldenburg, Medical Campus University of Oldenburg, Germany; 2 Dubai-London Specialty Hospital, Jumeirah, Dubai, United Arab Emirates

**Keywords:** vaginal surgery, müllerian ducts, abnormalities, Mayer-Rokitansky-Küster-Hauser syndrome, neovagina, reconstructive surgical procedures, methods, surgery of the female genitalia

## Abstract

Vaginal agenesis is a congenital anomaly that affects the life of one of each four thousand women around the world. There is a trend that patients request immediate surgical correction, instead of passive vaginal dilatation. Therefore a differentiated counselling should be provided.

We present a comparative chart, based on published evidence, with aspect to the available techniques, which will facilitate the decision-making process in the clinical practice.

From our point of view, the best results are achieved with techniques that combine the advantages of the minimal-invasive surgery with those derived of the use of peritoneum as covering tissue of the neovagina. Nevertheless there is a lack on interdisciplinary consensus about the best option to restore the physical and sexual quality of life.

## Zusammenfassung

Die vaginale Agenesie ist eine kongenitale Anomalie, welche in einer Frequenz von 1:4.000 auftritt. Die Patienten tendieren zu einer sofortigen chirurgischen Korrektur statt einer passiven Scheidendilatierung. Zur Entscheidungsfindung sollte eine differenzierte Begleitung vorgehalten werden.

Es wird eine evidenzbasierte, vergleichende Tabelle vorgestellt mit Darstellung sämtlicher zur Verfügung stehender Techniken zur Vereinfachung des Entscheidungsfindungsprozesses im klinischen Alltag.

Unserer Meinung nach werden die besten Ergebnisse mit Techniken erreicht, welche die Vorteile der minimal-invasiven Chirurgie kombinieren mit denen, die das Peritoneum als Neovagina-Gewebe nutzen. Trotzdem besteht ein deutliches Defizit in Bezug zu dem interdisziplinären Konsens für die adäquateste Therapie, um das physische und sexuelle Körperbild wiederherzustellen.

## Introduction

Since the first descriptions made by August Franz Karl Mayer, Carl Freiherr von Rokitansky, Hermann Küster and Georges André Hauser between 1829 and 1861 [1], there were many steps forward done on understanding, counselling and treatment of women with Mayer-Rokitansky-Küster-Hauser syndrome (MRKH) and comparable types of vaginal agenesis. It is known that any disruption, occurred during the development of the female genital tract between the fourth and twentieth weeks of gestation, leads to a failure in the fusion and canalization process of the müllerian ducts, urogenital sinus and sinovaginal bulbs. As a result, different grades of urogenital abnormalities are seen in women with normal karyotype 46, XX [2], [3]. Actually there is no a consensus about the best surgical approach to create of a functioning vagina, despite of advances in minimal-invasive surgery and in the sexual and reproductive rights of women to have access to best health care.

Classically, the genetic explanation to the female congenital genital tract disorder was attributed to a failure in the SRY-gen expression and a lack of activation of its downstream signaling pathways, but recently other mechanisms are described. A lack of expression of WNT4-, RSP01-, FOXL2- and TBX6-genes have been associated with deletion in 16p11.2 and 17q12, where at least 26 genes involved in the müllerian ducts differentiation are located. Additionally, an alteration in the apoptosis of the regulatory protein Bcl-2 is associated with the failure in regression of the uterine septum. Also, some toxic agents like thalidomide, diethylstilbestrol and radiation exposure have been associated with female tract defects [4]. 

In addition, any genital tract defect in women is an important fact to consider during reproductive counseling. For example, a woman could satisfy her desire to have genetically-related children thorough ovulation induction, egg retrieval and subsequent transfer to a surrogate uterus. 

The incidence of the vaginal dysgenesis is estimated between 1/4,000–1/10,000 women, 90% of them having MRKH [5]. This syndrome is characterized by normal functioning ovaries, normal external genitalia but vaginal aplasia (2–7 cm deep), and an absent cervix or rudimentary uterus. Some women (7–10%) have functional endometrium, and 25% have müllerian remnants [3]. MRKH can be associated with other müllerian (paramesonephric) ducts abnormalities, with two forms of clinical appearance. Type I: characterized by isolated absence of the proximal two thirds of vagina, and Type II or MURCS-association: a complex syndrome with müllerian duct aplasia, renal dysplasia and cervical somite anomalies [6]. Half of the patients with müllerian anomalies have associated malformations involving the upper urinary tract, abdominal wall, skeleton, heart or auditory system. Forty percent of women have pelvic kidney, horse kidney, unilateral agenesis, or duplication of the renal pelvis and ureter; 10–12% have rudimentary, supernumerary or wedge vertebrae. Some patients have conductive hear impairment due to middle ear malformations, mainly stapedial ankyloses, or heart defects like pulmonary valve stenosis, Fallot’s tetralogy and aorto-pulmonary window [5], [7]. Therefore, complementary diagnostic examinations should be performed to figure out the presence of concomitant abnormalities and to achieve a differential diagnosis, including androgen insensitivity, low-lying transverse vaginal septum and imperforate hymen.

As a part of the clinical assessment, conventional transabdominal, transrectal or translabial sonography is preferred to evaluate the urogenital anatomy. Magnetic resonance imaging (MRI) and laparoscopy are not routinely used in primary diagnosis. In cases of MRKH, the pelvic sonography shows normal ovaries and rudimentary uterine horns, or an absent uterus. MRI could be used to evaluate the presence of active endometrial tissue or suspected masses. Laparoscopy is recommended when ultrasonography is not conclusive or when a mass is found, offering the possibility to proceed with its extirpation [2], [4], [5].

Because of the normal external genitalia appearance, usually the diagnosis of vaginal agenesis is not made until adolescence or later during a regular visit, or when the woman complaints of amenorrhea, pelvic pain or difficulties to have intercourse. Specifically, in women with functional endometrium, cyclic or acyclic pelvic pain could be present as a sign of endometriosis or haematometra. The first as a consequence of retrograde menstruation, and the last could be accompanied by a painful mass [3], [5]. In our cohort of 53 women all patients complained from primary amenorrhea, with a mean age of 25 (13–40) years [6]. Consequently, obstetricians, pediatricians, general practitioners and midwives must do a complete physical examination of the newborn’s genitalia, as well as in the adolescent girl with primary amenorrhea, to make an early diagnosis of the genital abnormalities. 

Taking care of the psychological and cultural impact of vaginal agenesis on parents and women’s life is mandatory [8]. Because of its suspicion or diagnosis, emotional feelings could appear such as desperation, confusion, shame, incompleteness as a person and a woman, or rejection of the diagnosis. Especially, if the genital appearance is connected with psychic suffering, pressure or stress [9], [10]. We clearly need more evidence about the impact of this anomaly on the woman’s socio-cultural, and gender-related behavior patterns. 

## Methods

The aim of the treatment of vaginal agenesis is to create an anatomical and functional vagina that allows a satisfactory sexual life and psychological wellness. That is, the treatment must be guided by an expert group of professionals with an interdisciplinary and ethical approach [11] This means taking care of the girl’s and woman’s rights, preferences and informed decisions, in regard to the advantages, disadvantages, risks and benefits of available options. A neovagina should have 1) a correct axis canal of 2) adequate size and 3) secretory capacity to allow intercourse. For this purpose, two methods can be recommended: non-surgical passive self-dilatation of the rudimentary vagina, or surgical creation of a neo-vagina. 

Nonsurgical treatment is considered as first-line by pediatricians and some surgeons, in selected cases of a high-motivated girl or woman in the regular use of the dilatator [5]. The “Frank” procedure requires woman to manually place successive dilator devices in the vagina for 30–120 minutes per day. This produces progressive pressure, and invagination of the vaginal mucosa. A functional success, that is a 6–7 cm deep vagina, is achieved at a median of 19 months in many of them (70–95%). The “Ingram” procedure adds the use of a bicycle seat to create more pressure during the vaginal dilatation; more associated discomfort is reported. Both procedures require woman to have regular intercourse afterwards to avoid the retraction of the dilated canal [12]. 

The preference of the surgical approach depends upon the experience of the surgeon, instead of evidence or consensus [13]. Literature on neovagina procedures show a variety of described techniques, suchs as McIndoe, Davadon, Vecchietti and Creatsas vaginoplasty, or modified versions [14], [15], [16]. Nonetheless, there is a paucity of evidence about their long-time efficacy and patient satisfaction, due to the heterogeneity of pre-existing vaginal tissue, indications, population, and use of donor tissues. There is a lack of studies comparing all of the mentioned techniques [11], [17], [18], [19], [20], only some studies, reviews and metaanalyses exist, showing no superiority of any specific procedure and not adding new suggestions for neovagina creation or tissue-engineered autologous vaginal organs [21].

## Results

We have used the modified Vecchietti’s technique with good success [6], but the relatively equal results reported with all procedures for vaginal agenesis motivated us to make a clinical judgment in the very same way that a clinician does it at moment of counseling parents or women searching for a surgical treatment. In Table 1 (Tab. 1) we listed what we consider is the most relevant information around each procedure, offering an easy way to help the decision making process in the clinical practice. 

Vecchietti’s, Davadon’s, McIndoe’s, Adamyan’s or its modificated version? In our opinion the best results are achieved with techniques which combine the advantages of the minimal-invasive surgery with those derived of the use of peritoneum as covering tissue; as an alternative olive-traction dilatation is applicable. Obviously, the point of time to perform any of these procedures will depend on an adequate counselling process. But before accepting one of them as standard procedure for the creation of a neovagina in patients with MRHK, we need more evi-dence-based surgery to analyze the efficacy, risks, costs, and the bio-psycho-social impact of the proposed interventions.

New investigations should cover ethical, legal, medical, financial, psychological and cultural matters around this female genital defect such as: time of initiation and duration of use of vaginal devices, best age for vaginoplasty, waiting time until initiation of intercourse after surgery, patient’s satisfaction, quality of sexual life of the woman and her partner, psychological and cultural impact derived of the perception of the defect, secondary infertility, and the material used for the vaginoplasty. 

## Conclusions

In regards to vaginal agenesis management, there are satisfying techniques to create an anatomical and functional vagina, but there is a lack of evidence about a wide spectrum of matters that could potentially affect the quality of psychological and sexual life. Therefore, it is time to look for the best option in regard to important issues of women born with Mayer-Rokitansky-Küster-Hauser syndrome through prospective and possibly randomized studies comparing all plastic vaginal construction techniques, in a framework of sexual and reproductive rights of women with vaginal agenesis, including their access to advanced reproductive techniques. 

## Notes

### Competing interests

The authors declare that they have no competing interests.

## Figures and Tables

**Table 1:**
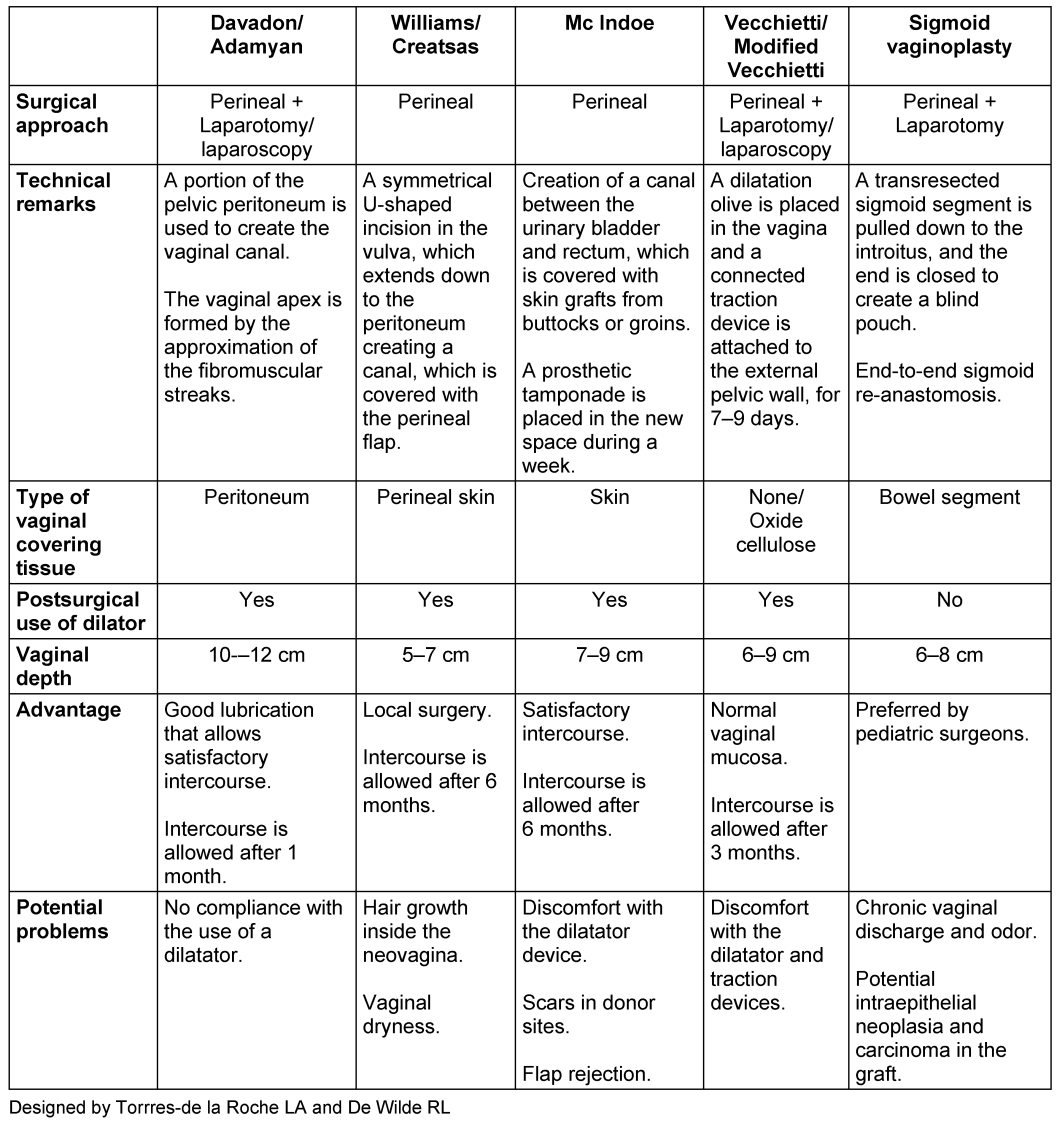
Counseling aspects of neovagina’s surgical procedures
